# Education Research: Development of a Workplace-Based Assessment to Enhance EEG Interpretation Skills for Medical Students

**DOI:** 10.1212/NE9.0000000000200349

**Published:** 2026-08-20

**Authors:** Hannah Kostan, Adam Dickey, Kareem Gadelmola, Alica M. Goldman, Vaishnav Krishnan, Lu Lin, Gabriela Tantillo, Atul Maheshwari

**Affiliations:** 1 Department of Neurology, Baylor College of Medicine, Houston, TX;; 2 Department of Neuroscience, Baylor College of Medicine, Houston, TX; and; 3 Huffington Department of Education, Innovation and Technology, Baylor College of Medicine, Houston, TX.

## Abstract

**Background and Objectives:**

Electroencephalography (EEG) interpretation is a core competency in neurology residency training, yet structured EEG education for interested medical students remains limited. Workplace-based assessments (WBAs) offer structured, formative feedback within authentic clinical settings and may address gaps in EEG education. We developed a novel EEG-specific WBA for use during a 2-week medical student “Epilepsy and EEG” elective to facilitate direct observation, structured feedback, and formative assessment of students. This prospective study evaluated the feasibility and perceived educational value of the tool, examined changes in faculty-reported medical student assistance over time, and characterized EEG features reviewed during WBA encounters.

**Methods:**

We conducted a prospective study during a 2-week Epilepsy and EEG elective at a single academic medical center (October 2024–February 2026). Students were asked to complete 4 EEG WBAs. Using a two-step model, students documented the verbal feedback and EEG features reviewed using a structured checklist before faculty completed a brief assessment using a 5-point entrustment scale. Faculty and students completed anonymous postintervention surveys assessing feasibility and educational value. Entrustment ratings were compared between Week 1 and Week 2 using the Fisher exact test.

**Results:**

A total of 20 medical students completed 79 EEG WBAs with 6 faculty members. The proportion of WBAs rated as requiring high levels of assistance (“I helped a lot”) decreased significantly from Week 1 to Week 2 of the elective (48.7% vs 21.1%, relative risk: 0.57, 95% CI 0.37–0.87, *p* = 0.017, Fisher exact test). Commonly reviewed EEG features included myogenic artifact (74.7%), eye blink artifact (69.6%), posterior dominant rhythm (55.7%), and sleep spindles (49.4%). The most frequent abnormal findings were focal epileptiform discharges (34.2%) and generalized epileptiform discharges (27.8%). Survey respondents (16 students, 5 faculty) reported high feasibility and educational value; no significant differences were observed between student and faculty responses.

**Discussion:**

As a proof of concept, these findings demonstrate that WBAs can serve as an effective formative assessment tool for EEG education in a brief elective for a subset of medical students. This model may also be adapted for neurology residents and fellows. Limitations include a single-institution sample, short elective duration, and multiple potential sources of variability.

## Introduction

Electroencephalography (EEG) interpretation is a core competency in neurology training and an essential clinical skill for the diagnosis and management of epilepsy and other neurologic disorders. The ACGME Neurology Milestones 2.0 include EEG interpretation under neurodiagnostic testing, reflecting its recognized importance for neurology residents and fellows.^1^ However, there is historically limited expectation for medical students to develop EEG interpretation skills, with a notable absence from AAMC Entrustable Professional Activity (EPA) 3 (“Recommend and interpret common diagnostic and screening tests”)^2^ and from the Core Curriculum Guidelines for a Required Clinical Neurology Experience.^3^ In addition, a recent scoping review of EEG instruction identified only 2 of 33 included studies as involving medical students, underscoring the paucity of evidence on EEG education at this level. Neither study involved workplace-based assessment (WBA) or longitudinal skill tracking. There is also no established consensus on which EEG features medical students should learn, further highlighting the need for descriptive data on what is taught in practice. Therefore, for medical students who are interested in learning more about EEG interpretation, exposure is often inconsistent and highly dependent on local curricula, with learning frequently occurring through passive observation rather than active skill development.

WBAs have become a central component of competency-based medical education (CBME) by enabling real-time, skill-specific feedback in authentic clinical settings.^4^ WBAs involve direct observation of clinical performance by a supervisor, followed by structured formative feedback and documentation of learner progression. Through repeated assessments, WBAs facilitate progressive entrustment, in which learners gradually assume greater independence as they demonstrate competence.^5^ However, implementation of WBAs can be challenging, particularly in subspecialty environments, because of concerns regarding faculty time constraints, evaluator burden, and workflow disruption.^6^ In the context of EEG education, prior instructional approaches have largely emphasized didactic or simulation-based modalities with knowledge-focused assessments, with relatively few interventions embedded within the clinical workplace or designed to track learner progression over time.

Given the limited evidence on EEG education for medical students and the absence of published WBA-based approaches for this population, there is a clear need to explore whether WBAs can be feasibly implemented and can capture meaningful learning in this context. Importantly, EEG education at the medical student level is typically offered as an elective for a subset of students with interest in neurology, rather than as a universal curricular requirement.

Although competency-based frameworks for EEG education have been proposed for neurology residents, including consensus lists of “must-know” EEG findings,^7^ there is limited evidence describing the use of EEG-focused WBAs at the medical student level. It remains unclear whether serial WBAs can capture meaningful changes in learner independence over a short clinical elective, whether such tools are acceptable to both students and faculty, and which EEG features are most emphasized during real-world teaching encounters.

WBAs may be particularly well suited to address these gaps because they allow direct observation of authentic EEG interpretation, structured feedback, and repeated assessment of learner independence over time. To evaluate this approach, we conducted a prospective pilot study assessing the implementation of an EEG workplace-based assessment within a 2-week medical student Epilepsy and EEG elective. The objectives of this study were to assess the feasibility and perceived educational value of the EEG WBA, examine changes in faculty-reported levels of medical student assistance over time, and characterize the EEG features most frequently reviewed during WBA encounters. By systematically evaluating this educational intervention, we aim to inform best practices for competency-based EEG education and provide a scalable model for formative assessment in neurology electives.

## Methods

### Study Design and Setting

We conducted a prospective pilot study evaluating the implementation of a WBA for EEG interpretation during a 2-week medical student Epilepsy and EEG elective at Baylor College of Medicine, designed to provide an immersive introduction to the epilepsy monitoring unit (EMU) and real-world EEG recordings. Students on the elective participate in EMU rounds, review bedside EEGs, and attend a once-weekly adult epilepsy clinic. The study was conducted between October 2024 and February 2026. The primary aims were to assess feasibility and perceived educational value of the EEG WBA, evaluate changes in faculty-reported levels of medical student assistance over time, and characterize the EEG features reviewed during WBA encounters.

### Participants

Medical students enrolled in the Epilepsy and EEG elective were eligible to participate. Students were in their second through fourth year of medical school and may or may not have completed a core neurology clerkship before enrollment in the elective. All students received an orientation with an introduction to EEG interpretation and given recommended reading to further their EEG interpretation skills. Participation in the EEG WBA was incorporated into the elective structure, and all students completing the elective during the study period were included. Faculty participants consisted of attending neurologists board-certified in clinical neurophysiology and/or epilepsy who supervised students during clinical EEG interpretation sessions. In cases where the primary attending was unavailable, a designated “WBA attending” (AM) completed the assessment by reviewing the EEG of a patient that the student was familiar with to ensure consistency and feasibility of implementation. The WBA attending was a faculty member with a specific interest in medical education who was designated for the study period and was available as a scheduled backup. Students were informed at the start of the elective about the WBA process and instructed to approach the on-service attending first; if that attending was unavailable, students contacted the WBA attending to arrange a review through Microsoft Teams based on mutual availability.

### EEG Workplace-Based Assessment Intervention

At Baylor College of Medicine, a two-step WBA framework has been successfully implemented in core clerkships to reduce evaluator burden while preserving educational value.^8^ In this two-step model, learners initially document the verbal feedback provided during the encounter, after which faculty complete a brief structured assessment using the 5-point entrustment scale described below. Building on this approach, we developed a novel EEG-specific WBA for use during a medical student “Epilepsy and EEG” elective. This tool was designed to facilitate direct observation of EEG interpretation within a competency-based framework, prompt targeted feedback, and document both EEG features reviewed and the degree of faculty assistance required using a standardized entrustment scale. Importantly, the assessment was explicitly formative and did not contribute to summative grading, encouraging honest feedback and learner engagement. Students were asked to complete a total of 4 EEG WBAs during the elective, with a target of 2 WBAs per week. During each WBA encounter, faculty directly observed the student interpreting a clinical EEG study and provided real-time verbal feedback. In a two-step model taking advantage of cocreation of narrative feedback,^9,10^ learners first documented the verbal feedback provided by faculty (eAppendix 1—Student Form), after which faculty completed a brief structured assessment^8^ (eAppendix 2—Faculty Form). Study data were collected and managed using REDCap electronic data capture tools hosted at Baylor College of Medicine.^11,12^

Students documented the EEG features reviewed during each encounter, including normal findings, normal variants, artifacts, and abnormal patterns, using a structured checklist informed by previously published EEG competency frameworks.^7^ Student documentation of EEG findings and verbal feedback occurred in separate fields within the same REDCap form. Faculty assessments used a standardized 5-point entrustment scale describing the degree of assistance required during EEG interpretation, with anchors reflecting progressive entrustment adapted from published entrustment frameworks^13,14^: “I had to do it,” “I helped a lot,” “I helped a little,” “I needed to be there but did not help,” and “I didn't need to be there at all.” Entrustment ratings were used solely for formative purposes and did not contribute directly to the student's summative grade.

### Data Collection

Data collected from the WBAs included the faculty entrustment rating and the EEG features identified during each assessment. In addition, students and faculty were invited to complete an anonymous post-intervention survey assessing feasibility and educational value of the EEG WBA. Survey items used Likert-scale responses to evaluate ease of use, perceived usefulness for learning EEG interpretation, adequacy of time to complete the assessment, and willingness to participate in the WBA even if it were not required. Free-text responses were also collected to capture qualitative feedback regarding the WBA experience (eAppendix 3—Survey).

### Outcomes

Primary outcomes included feasibility and perceived educational value of the EEG WBA as assessed by survey responses from students and faculty (Kirkpatrick Level 1) and changes in entrustment over time (Kirkpatrick Level 2).^15^ Secondary outcomes included distribution of EEG features reviewed across WBA encounters and rapid qualitative analysis of free-text responses.^16^

### Statistical Analysis

Descriptive statistics were used to summarize participant characteristics, survey responses, and the frequency of EEG features identified during WBAs. Changes in the proportion of WBAs in which faculty reported higher vs lower levels of trainee assistance over time were evaluated using the Fisher exact test. Comparisons between student and faculty survey responses were similarly analyzed using the Fisher exact test. Differences in the distribution of EEG features across serial WBAs were assessed using the Kruskal-Wallis test. Statistical significance was defined as a 2-sided *p* value <0.05.

Free-text survey responses were analyzed using a rapid qualitative analysis approach.^16^ For this analysis, 2 members of the research team independently reviewed all free-text responses and identified recurring themes using a structured summary template. Themes were compared and reconciled through discussion until consensus was reached. Given the small volume of free-text data, formal saturation assessment was not performed.

### Standard Protocol Approvals, Registrations, and Patient Consents

This study was conducted as an educational quality improvement initiative and approved by the Baylor College of Medicine Institutional Review Board (Protocol #H-56824). The EEG WBA assessments were formative, anonymous survey responses were collected, and no data were used for summative evaluation of students. Participation did not affect course grading or academic standing.

### Data Availability

Deidentified data from this study are available from the corresponding author on reasonable request, subject to institutional review board approval. Given the small sample size and single-institution setting, data are not publicly posted to minimize reidentification risk.

## Results

### Participants and WBA Completion

A total of 20 medical students (2 MS2s, 12 MS3s, and 6 MS4s) and 6 faculty members participated in the study. Across the study period, students completed 79 EEG WBAs during the 2-week Epilepsy and EEG elective. Each student completed a median of 4 WBAs (range 3–5), consistent with the elective design. The attending on service was used for the majority (65.8%) of assessments, with the backup “WBA attending” being used over Microsoft Teams the remainder of the time (34.2%).

### Change in Faculty-Reported Level of Medical Student Assistance

**Figure 1:**
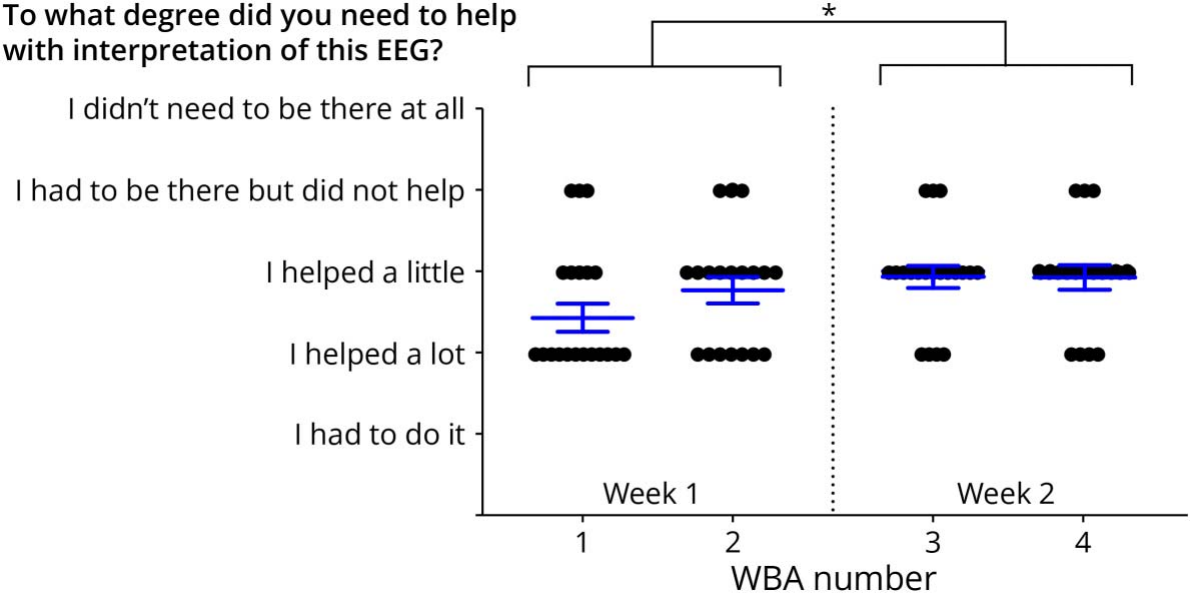
Progression of Entrustment Across Serial EEG Workplace-Based Assessments Stepwise increase in faculty-reported entrustment with completion of serial EEG WBAs. Faculty ratings demonstrate decreasing levels of required assistance across 4 sequential WBAs (mean ± SEM). A significant reduction in WBAs rated as “I helped a lot” was observed from week 1 to week 2 (n = 79 WBAs completed by 20 students and assessed by 6 faculty, *p* = 0.017, Fisher exact test). WBA = workplace-based assessment.

Faculty used a 5-point entrustment scale to document the degree of assistance required during EEG interpretation. Across serial WBAs, there was a stepwise improvement in faculty-reported entrustment, with decreasing levels of required assistance over time (Figure 1). Specifically, the proportion of WBAs in which faculty reported “I helped a lot” decreased significantly from Week 1 to Week 2 of the elective (48.7% vs 21.1%, relative risk: 0.57, 95% CI: 0.37–0.87, *p* = 0.017, Fisher exact test).

### EEG Features Reviewed During WBA Encounters

Across the 79 WBAs, a broad range of EEG features were reviewed and documented. The most identified findings were myogenic artifact (74.7% of all WBAs), eye blink artifact (69.6%), posterior dominant rhythm (55.7%), and sleep spindles (49.4%).

**Figure 2:**
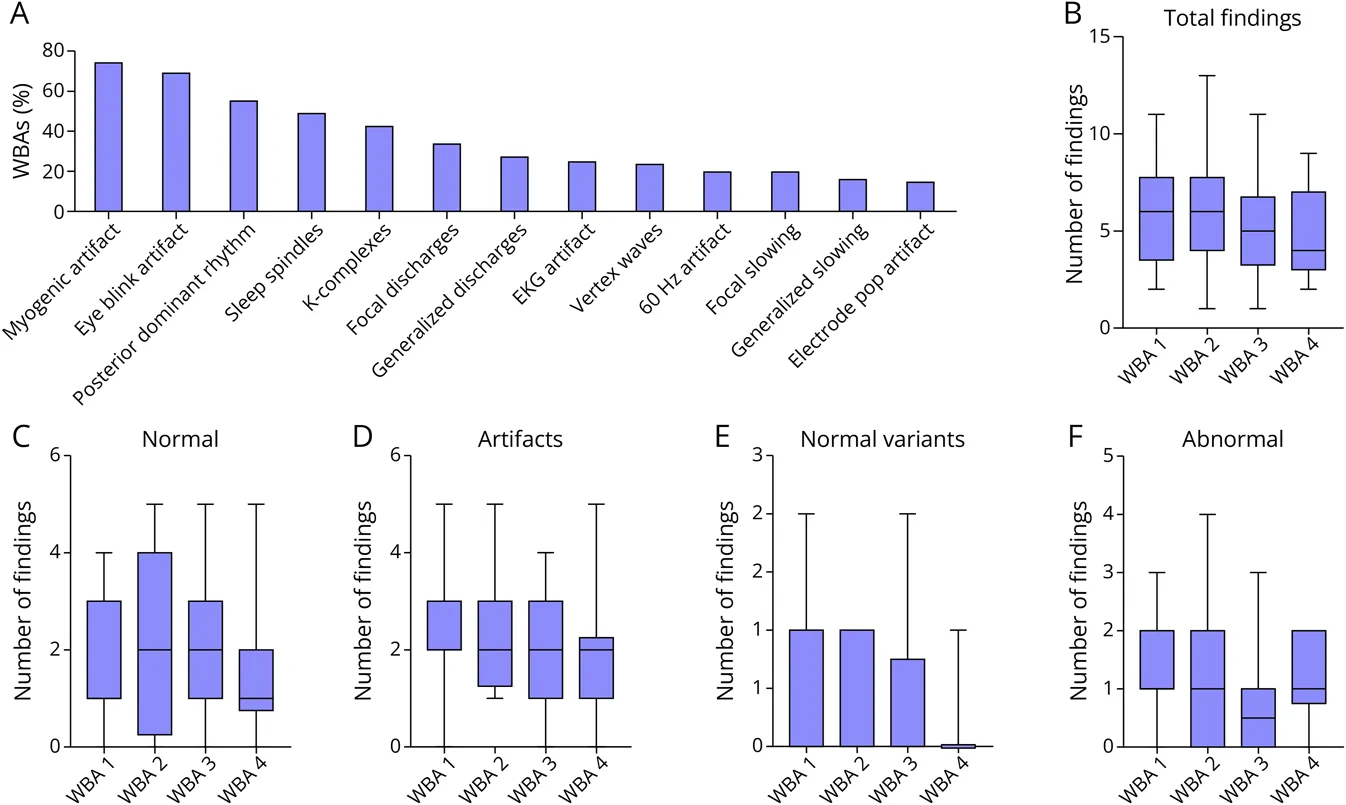
Distribution of EEG Features Reviewed During Workplace-Based Assessments (A) Frequency of EEG features identified across WBAs. The most commonly reviewed findings included myogenic artifact, eye blink artifact, posterior dominant rhythm, and sleep spindles. Abnormal findings were less frequent, with focal epileptiform discharges being the most identified abnormality. Less common normal variants were rarely reviewed. (B) Total number of findings as well as (C) Normal Findings, (D) Artifacts, (E) Normal Variants, and (F) Abnormal Findings were equally represented across all 4 WBAs (n = 79 WBAs completed by 20 students and assessed by 6 faculty, *p* > 0.05, Kruskal-Wallis test). WBA = workplace-based assessment.

Abnormal EEG findings were less frequently reviewed but were still represented across WBAs. The most common abnormality identified was focal epileptiform discharges (34.2% of assessments), generalized epileptiform discharges (27.8%), focal slowing (20.3%), and generalized slowing (16.5%, Figure 2A). Less common normal variants, such as mu rhythm, wickets, and small sharp spikes, were infrequently reviewed (<10% of WBAs). The distribution of total findings, normal findings, artifacts, normal variants, and abnormal findings were overall unchanged across WBA number (n = 79 WBAs completed by 20 students and assessed by 6 faculty, *p* > 0.05, Kruskal-Wallis test, Figure 2, B–F).

### Feasibility and Educational Value Survey

**Figure 3:**
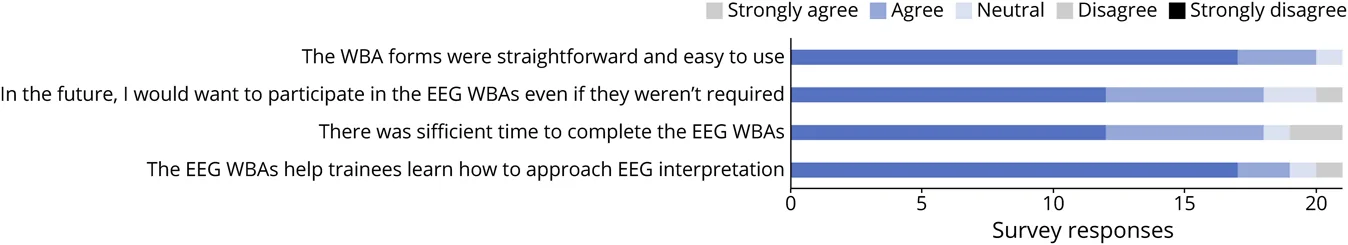
Feasibility and Educational Value of the EEG Workplace-Based Assessment Survey responses from 16 students and 5 faculty evaluating feasibility and educational value of the EEG WBA. Most respondents agreed or strongly agreed that the WBA was easy to use, contributed to learning EEG interpretation, allowed sufficient time for completion, and would be desirable even if not required. No significant differences were observed between student and faculty responses. WBA = workplace-based assessment.

A total of 21 participants (16 students and 5 faculty) completed the postintervention survey evaluating feasibility and educational value of the EEG WBA, not including the designated WBA attending (AM). Overall, responses indicated high acceptability and perceived educational benefit; both most of the students and most of the faculty agreed or strongly agreed to each question (eFigure 1). There were no statistically significant differences between student and faculty survey responses across each survey item (*p* > 0.05 for all comparisons, Fisher exact test), so data were aggregated for further global analysis (Figure 3). Most respondents agreed or strongly agreed that the EEG WBA was easy to use (95.2%), helpful for learning EEG interpretation (90.5%), and that there was sufficient time to complete the assessment (85.7%). In addition, 85.7% of respondents reported that they would want to participate in the EEG WBA even if it were not required.

### Qualitative Feedback From Students and Faculty

Free-text comments from students and faculty were reviewed using a rapid qualitative analysis approach to identify recurring themes related to the educational value and feasibility of the EEG WBA. Review of the comments revealed several consistent themes reflecting perceived benefits, challenges, and suggested improvements, summarized in Table.

**Table:** Themes Identified From Student and Faculty Free-Text Comments on the EEG Workplace-Based Assessment

Theme	Description	Representative quotations
Reinforcement of learning through reflection	WBAs supported reflection and reinforced learning by requiring students to document feedback and articulate understanding	“I was very impressed when the student wrote what he learned and understood… I felt that by the students verbalizing and writing down what they understood will reinforce it.” (Faculty) “In knowing that I was going to review one with an attending, I was motivated to keep up with my self-studying so that the review could point out my weak and strong areas.” (MS3)
Dedicated time for individualized teaching	WBAs created structured opportunities for focused, one-on-one EEG teaching that might not otherwise occur	“The WBA provided a useful, structured review time with attendings who otherwise may have been too busy to informally review.” (MS4) “I think the WBA was very useful as a way of establishing a guaranteed time to go over EEGs in a purely educational capacity.” (MS3)
Increased motivation and engagement	Anticipation of WBAs motivated learners to prepare and engage more actively with EEG interpretation	“In knowing that I was going to review one with an attending, I was motivated to keep up with my self-studying.” (MS3) “I find this learning tool a good motivator that improves student engagement in learning EEG basics.” (Faculty)
Logistical and psychosocial barriers	Some students experienced stress or discomfort related to obtaining WBAs and approaching busy faculty	“Four WBAs was slightly stressful to obtain in the timeframe.” (MS3) “It can be very uncomfortable to ask a busy attending to take the time to perform one.” (MS3)
Suggestions for improvement	Participants offered practical suggestions to improve feasibility and educational impact	“I would suggest going down to one per week.” (MS3) “Having some material or videos beforehand with specific goals for each session would be beneficial.” (MS3) “I would prefer to have the attendings fill out the WBA form in real time.” (MS4)

Abbreviation: WBA = workplace-based assessment.

Review of free-text comments identified recurrent themes related to reinforcement of learning, structured feedback and accountability, faculty–learner engagement, and perceived feasibility. Representative quotations from both students and faculty are provided to illustrate each theme.

Participants frequently described the WBA as reinforcing learning through structured reflection and documentation of feedback. Faculty noted that having students record feedback helped confirm learner understanding and promoted reflection, while students reported that the process reinforced key concepts and learning points. Another prominent theme was the role of the WBA in creating dedicated, individualized teaching time. Students highlighted that WBAs facilitated focused, one-on-one EEG review with faculty that might not otherwise occur in busy clinical settings.

Increased motivation and engagement with EEG learning was also commonly reported. Both students and faculty noted that the anticipation of reviewing EEGs during WBAs encouraged self-directed learning and preparation, contributing to greater learner confidence and reduced anxiety surrounding EEG interpretation. Several faculty members perceived WBAs as helping students overcome an initial fear of EEG interpretation.

At the same time, students identified logistical and psychosocial barriers to WBA completion. Some learners described discomfort initiating assessments with busy faculty and perceived stress associated with completing 4 WBAs within a short elective. Finally, participants offered constructive suggestions to improve the structure of the intervention, including adjusting WBA frequency, providing preparatory materials with clear learning goals, and modifying workflow to allow faculty to complete assessments in real time. Representative comments illustrating each theme are provided in Table.

## Discussion

In this prospective pilot study, we evaluated the implementation of a novel WBA for EEG interpretation within a 2-week medical student Epilepsy and EEG elective. Our findings demonstrate that the EEG WBA was feasible, well-accepted by both students and faculty (Kirkpatrick Level 1, referring to participant reactions and satisfaction as defined in Kirkpatrick four-level model of training evaluation), and capable of capturing progressive changes in learner independence (Kirkpatrick Level 2, referring to learning and skill acquisition) over a short clinical rotation.^15^ Together, these results suggest that WBAs represent a promising approach for formative assessment of EEG interpretation skills in medical student education and serve as a proof of concept for WBA-based EEG instruction that may be adapted for other learner populations, including neurology residents and fellows.

A key finding of this study was the observed improvement in faculty-reported entrustment over time, reflected by a significant reduction in the proportion of WBAs requiring high levels of assistance from Week 1 to Week 2 of the elective. This stepwise increase in entrustment supports the ability of serial WBAs to capture meaningful changes in learner performance as seen in clerkships with EPAs,^17^ despite occurring over a relatively brief clinical experience. Importantly, these assessments were embedded within routine clinical EEG interpretation rather than simulated or didactic environments, underscoring the value of direct observation in authentic practice settings.

The distribution of EEG features reviewed during WBA encounters further supports the educational validity of this intervention. Faculty teaching encounters most frequently emphasized core EEG features, including common artifacts, posterior dominant rhythm, and sleep architecture, as well as clinically salient abnormalities such as focal and generalized epileptiform discharges. Less common findings were reviewed infrequently likely because of reduced availability in the authentic clinical setting as well as faculty prioritization of foundational and high-yield concepts. This pattern aligns with a CBME framework that emphasizes focusing on outcomes, emphasizing abilities and promoting learner-centeredness.^4^ Given the absence of a standard consensus on which EEG features medical students should learn, these descriptive data may serve as a starting point for developing a core EEG curriculum for medical students in elective settings.

Our results directly address several gaps identified in a recent scoping review of EEG instruction by Fernandez et al.,^18^ which highlighted a predominance of knowledge-based assessments, limited use of workplace-based or longitudinal assessment strategies, and a relative lack of educational theory underpinning EEG instructional interventions. Notably, only 2 of 33 studies in that review involved medical students, neither of which used WBA or longitudinal assessment. By contrast, the EEG WBA described in this study emphasizes direct observation, formative feedback, and progressive entrustment within a CBME framework. By focusing on learner progression rather than isolated knowledge acquisition, this intervention advances the precision of EEG assessment beyond traditional pretesting and post-testing models.

The high feasibility and acceptability of the EEG WBA among both students and faculty further strengthen the case for its broader implementation. Survey responses indicated that participants found the tool easy to use, educationally valuable, and worth participating in even if not required. Notably, there were no significant differences between student and faculty perceptions, suggesting shared recognition of the WBA's utility. However, a substantial proportion of WBAs (34.2%) were completed with the designated WBA attending. This is particularly relevant given longstanding concerns regarding faculty burden associated with workplace-based assessments.^6^ The high rate of backup utilization likely reflects clinical demands on the on-service attending. Other institutions have found that dedicated expert assessors are often necessary to facilitate timely completion of WBAs.^19,20^ In addition, the two-step WBA model used in this study where learners document feedback before faculty complete a brief structured assessment may represent an effective strategy for mitigating assessment burden while preserving educational benefit.

Free-text comments from both students and faculty reinforced the quantitative findings, with participants describing the WBA as promoting reflection, increasing engagement, and creating structured teaching time, while also acknowledging logistical challenges such as time constraints and learner discomfort initiating assessments with busy faculty. Analysis of narrative content within the WBA forms themselves (e.g., length/specificity of comments, alignment between narrative and numeric scores, inclusion of learning recommendations) was not performed in this pilot study but represents an important future direction.

Several limitations should be considered when interpreting these findings. First, this was a single-institution pilot study with a modest subset of medical students with an interest in neurology, which may limit generalizability. Second, the short duration of the elective constraints conclusions regarding long-term skill retention or transfer to independent clinical practice. Third, sources of variability included differences in faculty judgment, varying medical student levels, and differences in difficulty of EEG being reviewed. Notably, entrustment ratings in this study may seem higher than expected for medical students early in a rotation. This likely reflects the focused, faculty-directed nature of the WBA encounters: rather than evaluating a student's ability to independently interpret an entire EEG from start to finish. Faculty typically selected specific features for students to identify during a brief, supervised review session (e.g., recognizing an eye blink artifact or identifying epileptiform discharges). As a result, entrustment ratings often captured performance on discrete tasks rather than comprehensive EEG interpretation. This distinction is important for interpreting the validity of these entrustment decisions and for future adoption of this assessment approach because the meaning of the scores depends on shared understanding of the scope of what is being assessed. We acknowledge that the scores reported here reflect growth in task-specific performance under direct supervision, and further work is needed to determine whether these gains translate to the achievement of overall intended outcomes for a medical student. Differences in how individual faculty calibrate their ratings, particularly regarding how much guidance constitutes “a lot” vs “a little” help, may further contribute to variability. Future implementations should consider providing faculty calibration guidance, feature-specific entrustment ratings, and explicit anchors for each entrustment level to improve inter-rater reliability. In addition, faculty were not blinded to the student's time on the elective, which may have contributed to higher ratings in the second week of the elective. Finally, while survey responses suggested high acceptability, qualitative data were limited and may not fully capture nuanced perceptions of the WBA experience.

Despite these limitations, this study offers important implications for EEG education. By demonstrating that a workplace-based, entrustment-focused assessment can be feasibly implemented and capture learner progression, our findings support a shift toward more authentic and longitudinal approaches to EEG assessment, with WBAs as the prototypical method of assessment at the top of Miller Pyramid of learning.^21^ As a proof of concept, the results of this study may transfer beyond the medical student context. We plan to evaluate the adaptation of this WBA for neurology residents and fellows, for whom EEG interpretation is a formally expected competency. WBAs may be particularly well suited for aligning instructional goals, assessment strategies, and feedback in EEG education, where structured exposure is often limited. Future studies should also evaluate the reliability and validity of EEG WBAs across institutions, explore their integration into longer rotations, and examine associations between WBA performance and downstream clinical behavior and outcomes (Kirkpatrick Levels 3 and 4, which refer to behavioral change in practice and patient-level results, respectively).^15^

This prospective pilot study demonstrates that a workplace-based assessment for EEG interpretation is feasible, well received, and capable of capturing progressive growth in entrustment during a medical student Epilepsy and EEG elective. By embedding assessment within authentic clinical EEG interpretation and emphasizing formative feedback and entrustment, the EEG WBA offers a practical approach to competency-based EEG education for a subset of medical students with interest in neurology. As a proof of concept, these findings support the potential adaptability of this model across training levels. These findings support the use of WBAs as a scalable strategy to enhance EEG instruction and assessment, particularly in areas where traditional knowledge-based evaluations predominate. Future work should evaluate the reliability and generalizability of this approach across institutions and learner populations and explore its role in longitudinal EEG training pathways.

## Glossary

CBME: competency-based medical education
EMU: epilepsy monitoring unit
EPA: Entrustable Professional Activity
WBA: workplace-based assessment
